# The Optimization of an eHealth Solution (Thought Spot) with Transition-Aged Youth in Postsecondary Settings: Participatory Design Research

**DOI:** 10.2196/jmir.8102

**Published:** 2018-03-06

**Authors:** Nicole VanHeerwaarden, Genevieve Ferguson, Alexxa Abi-Jaoude, Andrew Johnson, Elisa Hollenberg, Gloria Chaim, Kristin Cleverley, Gunther Eysenbach, Joanna Henderson, Andrea Levinson, Janine Robb, Sarah Sharpe, Aristotle Voineskos, David Wiljer

**Affiliations:** ^1^ Education Centre for Addiction and Mental Health Toronto, ON Canada; ^2^ McCain Centre for Child, Youth & Family Mental Health Centre for Addiction and Mental Health Toronto, ON Canada; ^3^ Department of Psychiatry University of Toronto Toronto, ON Canada; ^4^ Faculty of Nursing University of Toronto Toronto, ON Canada; ^5^ Institute of Health Policy, Management and Evaluation University of Toronto Toronto, ON Canada; ^6^ Centre for Global eHealth Innovation University Health Network Toronto, ON Canada; ^7^ Early Intervention Clinic Centre for Addiction and Mental Health Toronto, ON Canada; ^8^ Health and Wellness Centre University of Toronto Toronto, ON Canada; ^9^ QoC Health Toronto, ON Canada; ^10^ Slaight Family Centre for Youth in Transition Centre for Addiction and Mental Health Toronto, ON Canada; ^11^ Education, Technology & Innovation University Health Network Toronto, ON Canada

**Keywords:** students, transition-aged youth, mental health, substance use, eHealth, mobile apps, participatory action research, help-seeking

## Abstract

**Background:**

Seventy percent of lifetime cases of mental illness emerge before the age of 24 years, but many youth are unable to access the support and services they require in a timely and appropriate way. With most youth using the internet, electronic health (eHealth) interventions are promising tools for reaching this population. Through participatory design research (PDR) engagement methods, Thought Spot, a Web- and mobile-based platform, was redeveloped to facilitate access to mental health services by transition-aged youth (aged 16-29 years) in postsecondary settings.

**Objective:**

The aim of this study was to describe the process of engaging with postsecondary students through the PDR approaches, with the ultimate goal of optimizing the Thought Spot platform.

**Methods:**

Consistent with the PDR approaches, five student-led workshops, attended by 41 individuals, were facilitated to obtain feedback regarding the platform’s usability and functionality and its potential value in a postsecondary setting. Various creative engagement activities were delivered to gather experiences and opinions, including semistructured focus groups, questionnaires, personas, journey mapping, and a world café. Innovative technological features and refinements were also brainstormed during the workshops.

**Results:**

By using PDR methods of engagement, participants knew that their ideas and recommendations would be applied. There was also an overall sense of respect and care integrated into each group, which facilitated an exchange of ideas and suggestions.

**Conclusions:**

The process of engaging with students to redesign the Thought Spot platform through PDR has been effective. Findings from these workshops will significantly inform new technological features within the app to enable positive help-seeking behaviors among students. These behaviors will be further explored in the second phase that involves a randomized controlled trial.

## Introduction

### Background

The transition between childhood and adulthood can be difficult and many transition-aged youth will seek information about mental health and wellness (for the purposes of this study, we define transition-aged youth as those aged 16-29 years). Within Canada, mental health is a significant concern for young adults, with rates of mood disorders (8%) and substance use disorders (12%) higher among 15- to 24-year-olds than any other age group [1]. A Canadian survey of youths’ Web-based resource preferences showed that 52% of respondents aged 16 years to 25 years had previously sought information about mental illness symptoms, 47% had sought information about treatment, and 24% had sought Web-based questionnaires or assessment tests related to mental health and substance use [2]. An Australian survey reported similar findings, with one-third of 18- to 25-year-olds reporting primarily depending on the internet for information about mental health or substance use problems [3]. Given the increased use of Web-based resources as sources of mental health information, electronic health (eHealth) platforms are effective and promising options for delivering reliable information and improving access to mental health and wellness services for transition-aged youth. This study focuses on transition-aged youth in postsecondary settings. The terms *postsecondary students* or *students* will be used throughout this paper to describe our target population.

### What Is Thought Spot?

Thought Spot is a crowdsourced digital platform (mobile- and Web-based) that aims to better enable transition-aged youth in postsecondary settings to seek and access mental health and wellness services. It was developed by the Centre for Addiction and Mental Health and the University of Toronto (UT), with partners’ Ryerson University (RU), the Ontario College of Art and Design, and ConnexOntario. The project was funded by the Ontario Ministry of Training, Colleges and Universities [4]. Thought Spot is a student-led project that prioritizes inclusion through steering committees, working groups, and focus groups. Postsecondary students were involved in initial decisions about the project name, logo, product design, and project management. Through cross-organizational collaboration between postsecondary students and project partners, Thought Spot became a platform that invites students to share their knowledge about services, discover wellness options in their area, and read reviews of services. Using an interactive and crowdsourced map, users are able to geo-locate mental health and wellness spots. All spots are categorized by the type of services offered, and users can apply filters to personalize their search. Evaluation data collected during the first phase of Thought Spot showed that students felt a sense of ownership over the product because of their contributions, gained knowledge in the areas of mental health and wellness, and developed new skills throughout their involvement that were transferable to their education and future careers [4]. These data helped to inform the second phase of the Thought Spot project, which will be discussed in this paper.

This study includes two new stages: (1) optimizing the Thought Spot platform by engaging with students; and (2) measuring the impact of Thought Spot on help-seeking behaviors of students in postsecondary settings through a randomized controlled trial [4]. The objective of this paper is to describe the participatory design research (PDR) methods used during the optimization phase of the project and summarize the results. This study explored how PDR should be employed in designing and optimizing mHealth interventions for student mental health, as well as a discussion of the utility of the various data-gathering techniques.

## Methods

### Participatory Action and Design Research

The optimization of Thought Spot was conducted using PDR methodologies while following some of the principles of participatory action research (PAR) [4]. PAR is “a social, collaborative learning process” [5] that involves an iterative process of engaging end users in reflection to provide a deeper understanding of their needs and experiences [5-7]. Participants involved in such projects are empowered to work alongside researchers as equal contributors [8]. The goal of PAR is to include all stakeholders throughout the entire process [9] and to work toward and implement solutions that target clearly defined problems [10]. In this case, participants were asked to work on a specific problem that had already been established through PDR methods and, therefore, there were limitations to the extent to which PAR was applied. Similar to PAR, PDR involves the target audience in codesigning the technologies that audience will use [4]. In eHealth research, a number of techniques can be used to implement PDR, including workshops, ethnography, prototyping, and user-design activities [9]. PDR is most effective when the design of the intervention is driven by the values of the stakeholders [9]. PDR is based on actively engaging participants to take an equal role in developing and designing a product or service around their own experiences [11]. Although the principles of PAR and PDR align, each methodology relies on slightly different techniques. For example, PDR focuses on the design of a product or technology [4,11], whereas PAR focuses more on the process of research [9].

Various techniques and tools are used to generate understanding of the experiences and needs of end users. Methods in this eHealth project included two-part discussions (a large group discussion followed by a small group activity), semistructured interviews, questionnaires, personas, journey maps, and world cafés. The existing literature on PAR and PDR informed the structure of our activities with participants.

### Recruitment

A total of 41 participants attended 5 workshops. Participants were current students or recent graduates from the UT, RU, and George Brown College (GBC). To encourage students with lived experience to participate, explicit wording on the recruitment poster was used: “students with lived experience of mental health and substance use are encouraged to participate.” Workshops took place between July and September 2016.

Numerous methods were used to recruit participants for our engagement workshops. The workshops were promoted through preexisting Thought Spot social media accounts: Twitter, Facebook, and Instagram. Recruitment flyers were posted on departmental boards at UT, RU, and GBC. Academic departments and student organizations were identified as potential recruitment sites, including departments of psychology and social work, and health and wellness centers. The existing connections within the Thought Spot student advisory group and the research team were also used to help recruit participants. Participants received a small honorarium and public transit tokens for attending each workshop, and food was served at all workshops. Participants also received a list of mental health and addictions resources they could access, if needed. All participants signed an informed consent form that provided an overview of the study objectives, risks, benefits, confidentiality, and contact information. This study obtained research ethics board approval from the Centre for Addiction and Mental Health, UT, RU, and GBC.

### Data Collection

#### Usefulness, Satisfaction, and Ease of Use Questionnaire

To assess participants’ opinions on the platform’s usefulness and satisfaction, the Usefulness, Satisfaction, and Ease of Use (USE) questionnaire was distributed to all participants [12] following each workshop. The USE questionnaire is a standardized scale consisting of 30 quantitative questions divided into 4 main sections: Usefulness, Ease of Use, Ease of Learning, and Satisfaction. Questions are asked using a 7-point scale from strongly disagree to strongly agree. The data collected informed the rebuild of the Thought Spot platform.

#### Codesign Workshops

A total of 5 workshops were planned based on the codesign activity methods identified through preliminary research. Each activity used different elements of PDR techniques to elicit information through a range of group formats (Table 1). Sociodemographic information and a postworkshop evaluation survey were collected at each workshop. All workshops were audio-recorded and flip chart notes collected. Workshops were facilitated by a research coordinator with 7 years of experience in facilitating focus groups with vulnerable populations, a research analyst with over 5 years of experience in facilitating focus groups through alternative methods of engagement, and 3 practicum students interested in cocreation. Facilitators used a semistructured question guide tailored for each activity to guide the discussion. Sample images from flip chart notes taken during the workshops can be found in Multimedia Appendix 1.

Various facilitation techniques were used in the workshops. A semistructured approach to facilitating group discussions established flexibility while maintaining an overall sense of direction throughout the conversation between participants [13]. All workshops were organized into 2 distinct parts. Two-part discussions allowed for an initial general conversation about the identified topic or question, followed by a more targeted conversation [14]. This approach gives participants the opportunity to discuss general topics outside the context of the product being researched. For example, in one of our two-part discussions, questions in the first portion of the discussion focused on the general experience of accessing mental health and wellness services as a student, without any focus on Thought Spot. Participants were then divided into smaller groups to further explore and discuss barriers to seeking help related to mental health and wellness in a more intimate setting. Mazzone et al reported that when engaging with youth, small groups “allow for greater focus on each task” while fostering creativity. Dividing participants into smaller groups during discussions helped ensure that most participants were able to contribute [15,16].

**Table 1:** Workshop descriptions.

Workshop #	# of participants	Structure	Purpose
1	6	Semistructured, two-part discussion	Explore the usage of eHealth apps and gain insights into the Thought Spot user experience from experienced users.
2	8	Semistructured, two-part discussion with small breakout groups	Explore the use of eHealth apps and gain insights into the Thought Spot user experience from new users.
3	8	Semistructured, two-part discussion using personas	Determine whether Thought Spot meets the health needs of its user personas.
4	6	User journey mapping followed by semistructured discussion	Explore the experiences of new users through journey mapping.
5	13	Focus group followed by a world café	Gather information on what health needs Thought Spot addresses, what features to include in its redesign, and what would keep users coming back.

Our team used personas, world café, and journey mapping as methods for gathering information from participants. Personas are “realistic descriptions of a type of client or user” that help to establish an understanding of the needs and perspectives of those for whom a product is being designed [17]. The process of walking through the experiences of users helps to guide and focus improvements for specific products or services [17]. Personas provide an appropriate amount of structure that allows participants to communicate ideas in the context of a larger topic in a tangible way [18,19].

In our study, several personas were developed to help capture diverse backgrounds and to outline different scenarios one might encounter when looking to access mental health or wellness services in the Greater Toronto Area (GTA). An example of one of our personas can be found in Multimedia Appendix 2. The use of personas maintained a level of confidentiality by inviting participants to discuss how Thought Spot could meet their needs without needing to disclose their own personal experiences.

Journey mapping was another technique used for collecting information. It is commonly used to evaluate the user experience through accessing and interacting with a service or product over time [17]. Suggestions for improving health interventions or accessing programs have been uncovered through journey mapping as participants highlight specific points of contact within the health care system and the emotions they experience while navigating it [11,17]. A world café exercise was used during the final Thought Spot workshop, which focused on confirming our findings from the previous four workshops and eliciting diverse perspectives. World cafés involve small groups cycling through a series of questions at different stations and building on the answers of the previous groups [20,21]. This method attempts to obtain diverse perspectives, rather than to achieve consensus, to better understand the overall experience of participants [20]. By dividing into small groups, participants had an additional opportunity to express their opinions about Thought Spot and share their overall experience of help seeking. Finally, participants who were uncomfortable discussing sensitive topics in a group setting could give written feedback through questionnaires [15].

#### Workshop Evaluation Feedback

Feedback surveys were completed by participants following each workshop. The surveys collected information on how participants heard about the workshop and what they liked and disliked about it, as well as asking participants whether they had any additional questions or comments about the project in general. This feedback was used to guide adjustments to subsequent workshops.

### Data Analysis

#### Usefulness, Satisfaction, and Ease of Use Questionnaire Analysis

IBM SPSS Statistics 24 was used to analyze the USE questionnaire data (N=27). Values for low (1-2), medium (3-5), and high (6-7) satisfaction in the USE questionnaire were calculated by taking the sum of responses for each question.

#### Inductive Content Analysis

The data collected during the workshops were analyzed using content analysis, a method often used “for making replicable and valid inferences from data to their context, with the purpose of providing knowledge, new insights, a representation of facts, and a practical guide to action” [22]. An inductive approach to content analysis was chosen, as no preconceived set of categories or framework was used to design the study [22]. This method is advantageous because the topics discussed came directly from participants [23].

The method of inductive content analysis involves 3 main phases: preparation, organizing, and reporting [24]. The preparation phase involves selecting the object of study for the content analysis, known as a unit of analysis [25]. Although the unit of analysis can come in many forms, whole interviews or observational protocols are most suitable [25]. The organizing phase involves the open coding of transcripts, generating a list of topics, and grouping similar topics together to form categories [24]. A process of abstraction then occurs, whereby a general description of the research topic is created from the groups of topics [24]. During the final, reporting phase, a model or conceptual map is generated to present the results [24].

Audio recordings of the workshops were sent to a professional transcriptionist. The transcripts were then anonymized and sent to all participants for review. The varied facilitation techniques (breakout groups, small/large group discussions) resulted in 16 transcripts produced from the 5 workshops. The units of analysis were transcripts of whole workshops. Transcripts of activities within each workshop were combined to create a single transcript for each workshop. Combining all transcripts from each workshop resulted in 5 discrete transcripts and ensured that data collected from each workshop were equally prioritized. To identify key discussion topics, 2 researchers independently coded a sample of 3 transcripts. A coding meeting was held where both researchers compared interpretations of the transcripts. The topics were compared and combined into categories of content topics in a coding matrix (Multimedia Appendix 3). Following the creation of the coding matrix, the 3 full transcripts were coded by each researcher to test its validity. Once the coding matrix was verified, content analysis of all transcripts was performed using QSR International NVivo 10 for Windows qualitative analysis software by one of the researchers.

## Results

### Demographics

In total, 41 students participated in the workshops: 29 females and 12 males. Most participants were aged between 19 and 24 years (Table 2). Most participants were full-time university or college students (n=39). Of all participants, 2 participants were attending school part-time. More than half of participants indicated that they had some experience with mental health or substance use concerns (Table 2).

### Usefulness, Satisfaction, and Ease of Use Questionnaire

**Figure 1:**
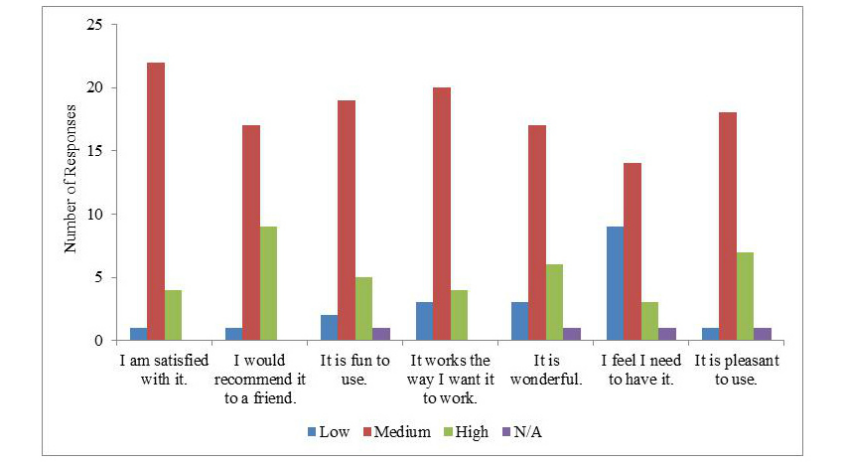
Participant responses to the satisfaction questions from Usefulness, Satisfaction, and Ease of Use Questionnaire. N/A: not applicable.

The majority of respondents indicated a medium level of satisfaction with original version of Thought Spot (Figure 1).

**Table 2:** Participant characteristics (n=41).

Participant characteristic		Percentage (%)
**Age (year)**		
	19-21	24
	22-24	63
	25-27	7
	28-29	3
	Other	3
**Experience with mental health and/or addiction issues**		
	Yes	54
	No	41
	Don't know	5

### Qualitative Codesign Workshop Data

**Figure 2:**
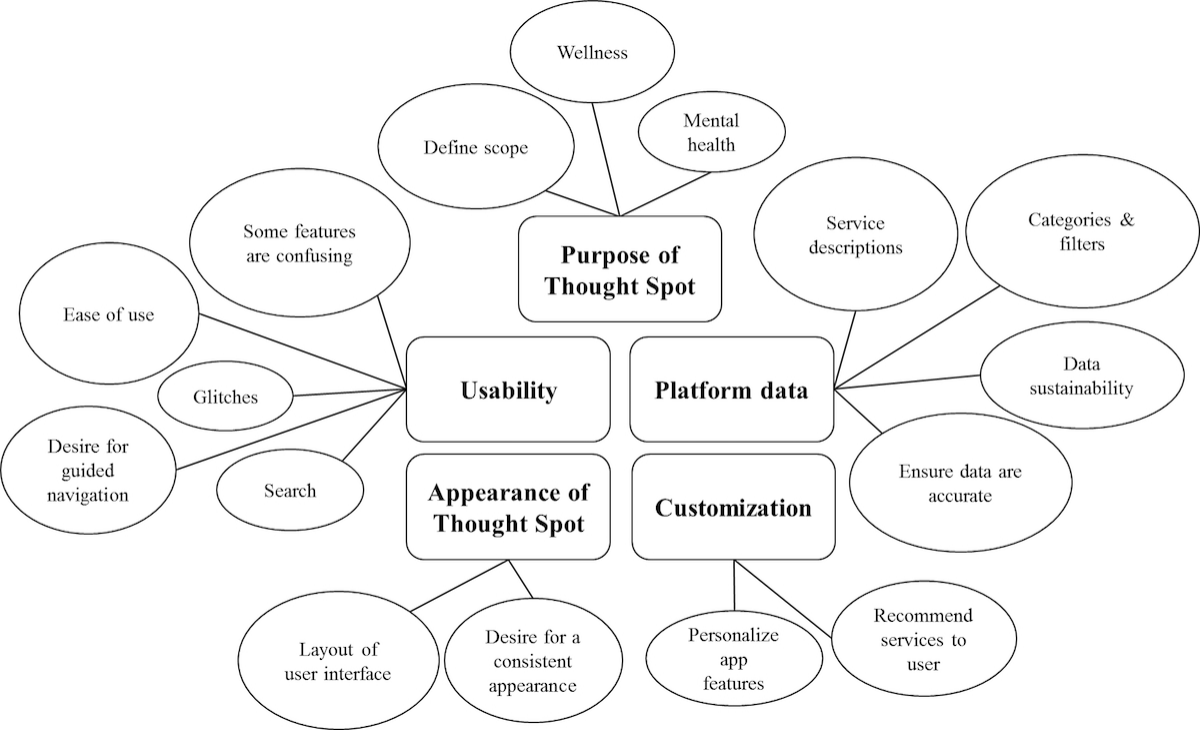
High-level conceptual map of topics discussed during workshops.

The results from the workshops were reviewed by the core research and design team to inform the redesign of Thought Spot. As each workshop focused on different topics and the facilitators used various data collection methods, the content that was coded varied. A high-level concept map was created based on the coding of topics across all transcripts (Figure 2). In Figure 2, larger circles represent topics that were discussed more often during the workshops.

### Purpose of Thought Spot

Participants often disagreed on the purpose of Thought Spot. Some thought that recreational programs, social clubs, and tips for maintaining mental well-being should be included in the platform. For others, however, limiting the focus to mental health services seemed fitting. Further discussions explored this difference in opinion, and workshop facilitators explained the purpose of Thought Spot as a wellness app that recognized the broad determinants of health, mental health, and wellness.

When discussing different types of wellness activities and services that could be included in Thought Spot, participants also mentioned preventative approaches such as connecting users to student social groups or building a function for tracking emotions, moods, and thoughts. One participant stated:

The best way to treat mental health is through mental wellness. Prevention is the best policy. That also speaks about some of the transitions; if you’re starting something new [like starting university or college], you might want to be able to have access to community services like yoga classes or support groups.

Participants who supported framing Thought Spot as a wellness app believed that this would increase its overall accessibility to a broader range of people. Although there was a general consensus on incorporating a wellness approach in the platform, participants encouraged the research team to carefully consider the inclusion criteria for these services.

### Usability

Usability was a concern for some participants. Certain features of Thought Spot were described as confusing and difficult to use. For example, adding a spot was particularly difficult for participants, as one participant explained:

I found it very difficult to try to add anything...I tried everything 3 times and it would freeze or shut down.

Participants mentioned that using the categories or filters to find services was challenging due to the confusing categorical structure or a lengthy list of filters. For example, they found the category “Health and Social Services” to be too broad because it included youth drop-in centers as well as community produce markets and community parks. Participants suggested including descriptions of categories and tutorials that walk users through each feature in the app. Some participants felt that, in general, the app was easy to use due to its similarity to other geo-location apps such as Google Maps and Yelp. One participant stated:

I think the way Google Maps does it is useful for me with the TTC [public transit] or walking routes, things like that.

Participants requested that the navigation feature of Thought Spot be expanded to include in-app directions to spots.

### Platform Data

Discussions about data varied with each workshop group. Topics included the categories and filters, crowdsourcing, description of services, missing information, forming partnerships, ratings, sustainability, and data verification. As Thought Spot is a crowdsourced app, participants identified active moderating as a method of maintaining a clean dataset. One participant suggested:

Anyone can add something, so maybe there could be a way to confirm that these are valid. With a checkmark or something that shows that this has been verified by someone on the back-end.

Participants recommended that a member from the research team with experience and interest in moderation and data cleansing be responsible for this process. This moderator would fill in missing information (eg, address, hours) and populate description fields. Participants requested that descriptions of spots include details such as cost, appointment or walk-in, accessibility, hours of operation, parking, and available languages.

### Appearance of Thought Spot

Discussions about the appearance of Thought Spot often focused on the layout. Some participants felt that the interface was too cluttered and overwhelming. For example, the resources page was described as “really dense” with “a lot of text and it’s just black and white, so it’s not pretty.” Most feedback was about having too much information displayed on each screen.

Generally, participants liked the overall consistency with the color schemes and design layout, but some participants highlighted inconsistencies in how the app is displayed on various devices (iPads, iPhones, Android). Participants agreed that a consistent use of color, shapes, and layout of features was ideal. Comments about the app’s color scheme were positive. 

Participants responded well to the “friendly and approachable color scheme.” In the words of one participant:

You have a lot of greens and softer lilacs and blues going on. It feels like a health and wellness app.

### Customization

Overall, participants were in favor of being able to customize Thought Spot to each user’s specific needs and preferences. The participants proposed the ability to save a list of favorite spots and the option to personalize different features. For example, some participants requested the ability to modify general settings such as sounds or number of push notifications they received. Participants also discussed the possibility of developing Thought Spot as a smart app, which would provide recommendations to users based on their unique needs, interests, and search history. Participants also recommended that suggested spots could be based on the user’s mood. One participant suggested:

Maybe just have an option that allows you to put in how you feel that day, or things that concern you that you might want to talk about. Then it will take in those things and suggest certain services or certain people that you can reach out to.

### Workshop Evaluation Feedback

After each workshop, participants were sent a short feedback survey containing 5 questions. These evaluations indicated that participants enjoyed the collaborative, interactive environment and felt safe sharing their thoughts and opinions. Our team received conflicting feedback about the duration of the workshops. For some participants, ensuring that adequate time was allocated to each portion of the workshops was a key concern, whereas others thought the workshops could have been shorter. Participants mentioned that time spent filling out surveys (sociodemographic and USE questionnaire) could have been better spent with group discussion, and that surveys could be filled out before the workshops. Of the 41 evaluation feedback surveys distributed, 25 were completed.

## Discussion

### Target Population Composition

Comparing our participants with Ontario postsecondary students in general, we see some similarities and differences. The majority of our participants were female (71%, 29/41), and 95% (39/41) were full-time postsecondary students compared with 55% and 80% for all Ontario students in the 2015 and 2016 school year, respectively [26]. Moreover, 87% (36/41) of our sample were between the ages of 19 and 24 years, whereas Statistics Canada reports that 46% of Ontario postsecondary students are between the ages of 20 and 24 years [27]. Finally, 54% (22/41) of participants indicated they had lived experience of mental health and/or substance use which is higher than the reported Canadian average for this population [2]. Given that this is a qualitative study that relied on self-selection of a small sample size, we did not anticipate recruiting a fully representative sample of our target population.

### Optimization of Thought Spot

Our team focused on eliciting qualitative and quantitative feedback on how to improve the first version of Thought Spot. There was an overall interest among participants in helping to develop an mHealth intervention that streamlines access to mental health and wellness services for their peers. Results from both the qualitative data analysis and USE questionnaire show a moderate level of satisfaction with the current Thought Spot platform. When assessing its usability, participants discussed the features they found confusing to use, ambiguity surrounding the categories and filters, and the desire to ensure that the information about services is accurate and up to date.

At times the feedback from students conflicted and therefore presented challenges for the project team to make design decisions. For example, during discussions about whether wellness-type services should be included in the platform, some students supported the idea, but others disagreed, wanting the platform to focus solely on mental health services. Conflicting opinions were taken into account during the redesign process. Cost, timelines, and capacity to implement some of the suggestions also had to be thoughtfully weighed by the project team.

To help guide the design process, a design working group was established that included research team members, technological partners, and student representatives. This group discussed and prioritized the needs and wants identified by the students who participated in the workshops. Design decisions were also brought back to the Thought Spot Student Group, our advisory group, for feedback and confirmation.

### Use of Participatory Design Research Methods

The success of this project to date supports the move toward PDR in the area of mHealth interventions targeted toward transition-aged youth [28]. Fundamental to PDR is the need to involve target users in all aspects of the research and to empower them to have a sense of ownership over the product. Although it may be difficult to include participants as equal members of the research team, efforts should be made to ensure that their views are valued and embedded into the product design whenever possible. The strength of these research methods lies in ensuring that an open collaboration between researchers and participants exists. Using PDR during this optimization phase of the project created an environment in which our participants were encouraged to contribute their experiences and ideas related to health, mental health, and wellness to ultimately improve the Thought Spot platform.

Motivating students to engage in PDR requires fostering a sense of understanding of the approach and allowing participants to engage in a way that maintains confidentiality and safety [19]. To address potential power imbalances between participants and researchers, the facilitators ensured that good communication and respect between these 2 groups were established at the beginning of each workshop [29]. Cocreating a series of workshop guidelines with participants was essential to ensure that the workshop environment was open, collaborative, and safe. Workshop evaluations indicated that participants enjoyed the collaborative, interactive environment, and that they felt welcomed to share their thoughts and experiences. The facilitators made a concerted effort to foster an environment where differing opinions and experiences were valued and encouraged by actively listening to each participant by facilitating discussion so all participants could contribute thoughts and ideas.

Various PDR methods were used to elicit feedback on participant’s opinions of Thought Spot from the perspective of postsecondary student’s experiences of mental health and wellness. The techniques found to be most useful during the Thought Spot workshops were small group discussions, persona exercises, and journey mapping. The use of personas was previously used by Nicholas and colleagues to help research participants discuss youth-specific challenges [19]. We found similar benefits when using this technique in that imagining a best outcome for the persona increased a sense of ownership and empathy in participants [19]. The use of journey maps during the workshops gave participants the opportunity to develop and express a narrative about their experiences with the platform. In addition to facilitating discussion of user experiences, the journey maps prompted discussion of the appearance and purpose of Thought Spot. The journey mapping exercise proved to be very effective in identifying areas for improvement. This could be due to the open-ended format of journey mapping, where participants were able to provide feedback that was not limited by questions asked by facilitators.

These creative techniques made the workshops accessible to participants, helped them relate to the topic of mental health, and encouraged them to contribute to solutions [19]. Collaborating with students in PDR has significant benefits, including establishing common ground and understanding the needs and motivations of the target population [19]. Each PDR technique used during the workshops assisted researchers in collecting helpful feedback for optimizing the Thought Spot platform. Understanding the unique strengths of each method to answer specific questions or collect different types of feedback is critical to the success of PDR. Thoughtful consideration of what techniques to use in a PDR project can help to ensure that the desired feedback is collected.

### Limitations

Participants represented only 3 postsecondary campuses in the GTA, all of which are located in downtown Toronto. The experiences of these students' mental health help seeking may vary from those studying and living in other parts of the city where services are less accessible. Although efforts were made to recruit more males, the majority of our participants were female, potentially skewing our data. Another challenge involves the methodology used in our study. Using various methods to gather information in each workshop meant that slightly different data were collected. In addition, a relatively small number of participants (N=25) completed the USE questionnaire, and descriptive data analysis was performed by the research team.

### Conclusions

Students encounter barriers to seeking help, such as confusion when navigating the health system and fears of being labeled. Services made available through a crowdsourced platform may facilitate and enhance the help-seeking process. Moderate satisfaction with the current Thought Spot platform can be improved by addressing concerns with usability, content accuracy, and customization. PDR methods are useful tools when engaging students in research related to eHealth. PDR is most effective when the design is driven by the values of the stakeholders [9]. The values expressed by students have guided Thought Spot’s platform optimization and redesign. Engaging with students through in-person workshops and activities was very effective for this project. The redesign of Thought Spot was guided by feedback received through these PDR workshops. Next steps include testing the effectiveness of the platform through a randomized controlled trial and continuing to enhance the overall project operations based on feedback received from student participants.

## Appendix

Multimedia Appendix 1 Example images from workshops.

Multimedia Appendix 2 Thought Spot persona example.

Multimedia Appendix 3 Coding matrix of topics discussed during Thought Spot workshops.

## Abbreviations

eHealth: electronic health
GBC: George Brown College
GTA: Greater Toronto Area
PDR: participatory design research
PAR: participatory action research
RU: Ryerson University
USE: Usefulness, Satisfaction, and Ease of Use
UT: University of Toronto
