# Supplementary material for: The Optimization of an eHealth Solution (Thought Spot) with Transition-Aged Youth in Postsecondary Settings: Participatory Design Research
Source: J Med Internet Res. 2018 Mar 6;20(3):e79. doi: 10.2196/jmir.8102 (PMC5861299; doi:10.2196/jmir.8102)

A. Flipchart notes from discussion of barriers to seeking help. (Workshop 1)

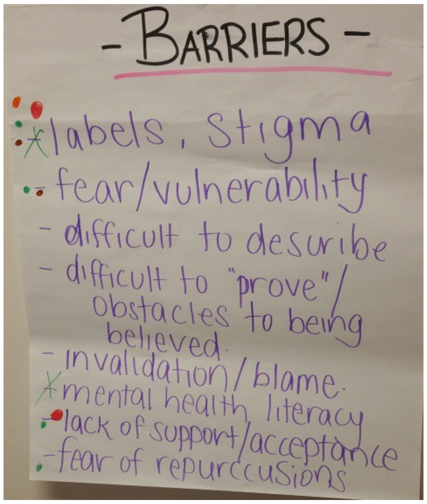

B. Flipchart notes from discussion of facilitators to seeking help. (Workshop 1)

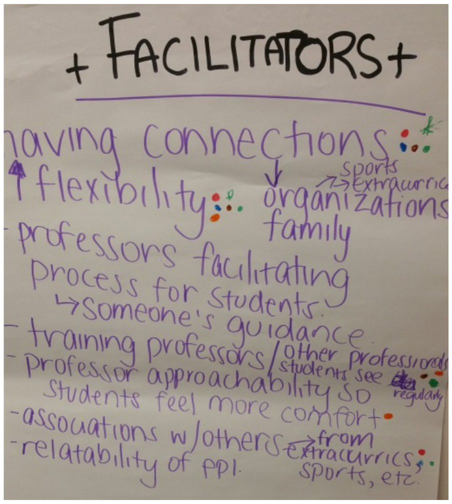

C. User journey map. (Workshop 4)

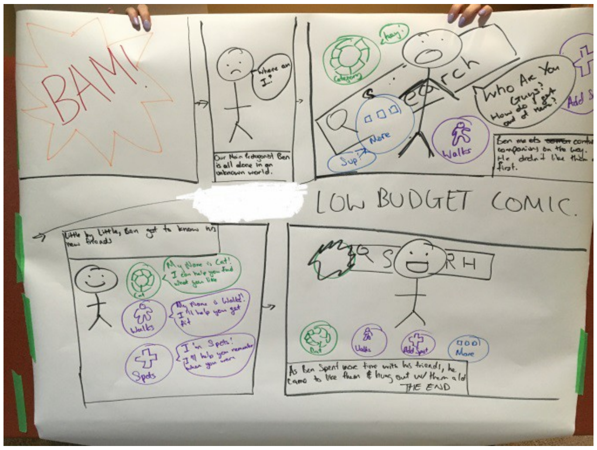

D. User journey map. (Workshop 4)

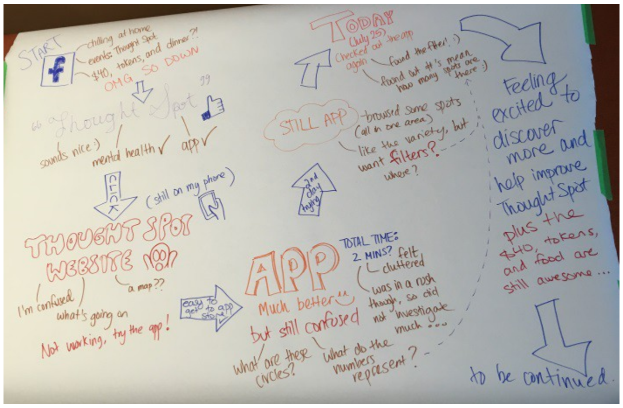

Supplement: Multimedia Appendix 1 [file jmir_v20i3e79_app1.pdf]
