# Supplementary material for: The Optimization of an eHealth Solution (Thought Spot) with Transition-Aged Youth in Postsecondary Settings: Participatory Design Research
Source: J Med Internet Res. 2018 Mar 6;20(3):e79. doi: 10.2196/jmir.8102 (PMC5861299; doi:10.2196/jmir.8102)
Supplement: Multimedia Appendix 2 [file jmir_v20i3e79_app2.pdf]

APPENDIX 2

Thought Spot Persona Example

ALEX—"The misunderstood youth"

GENDER: Male

GENDER IDENTITY: Cisgender

AGE: 17

SEXUALITY: Gay

RELIGIOUS FAITH: Atheist

CITIZENSHIP: Canadian

RELATIONSHIP STATUS: In a relationship

NUMBER OF DEPENDENTS: none

TYPE OF DISABILITY, IF ANY:
none

CURRENT UNIVERSITY:
OCAD University (Domestic student)

PROGRAM – FIELD OF STUDY:
Undergraduate – Integrated Media

YEAR OF STUDY: First year

PERSONALITY TRAITS
- Talkative
- Energetic
- Trusting
- Outspoken
- Verbose

LIVING ARRANGEMENTS
- Living off campus in an apartment in Kensington market
- Originally from a small town up north (Oro-Medonte)

FINANCIAL STATUS
- Not financially independent
- Family income level: middle
- Source of funds for educational expenses: Parents and OSAP

TRANSPORTATION ARRANGEMENTS
- Does not have a car
- Mostly walks or uses public transportation
- Walks to school

INVOLVEMENT IN CAMPUS ACTIVITIES
- Not involved (does not know about LGBT services or groups

TASKS
- School and homework obligations
- Making time to see his partner (also lives in the city)
- Looking for campus activities to be more involved in school

NEEDS
- Significant social support to keep from feeling alienated
- Suspected symptoms of depression and needs emotional support

BACKGROUND INFO
Alex is an undergraduate student at OCAD University studying Integrated Media. He struggles with strong social barriers because of his sexuality and feels alienated from both himself and the commu- nity at large. He loves to be around people but often fears feeling rejected and can be sensitive to others.

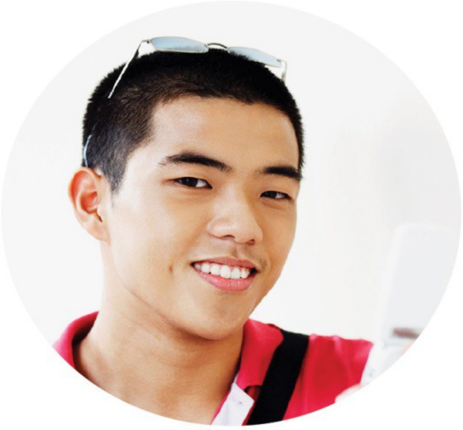

"It's hard to connect with people. When I'm anxious around people I tend to talk a lot but it usually annoys people more so in the end."

"They tell you that you should just be yourself, but what if yourself is someone that's repulsed by the community at large? Sometimes I wake up and don't know how to be both "me" and someone that people will like as well."

"My dad barely talks to me these days. Telling him I was gay was probably one of the most scarring experiences of my life; he was trying to hide his disappointment from me but it was just all over his face. It was horrible."

PROBLEMS
- Trouble adjusting from a rural to urban environment
- Making friends and fear of being judged from peers
- Anxiety around large groups of people
- Feels as though no one understands him
- Properly expressing his interests and himself
- Experiences discrimination, bullying, harassment, and marginalization.
- Concerned about physical safety
- Deals with frequent mood swings and low moods
- Rejected by his father over his sexual orientation → source of conflict
  - Bad relationship with father
  - Mother is supportive, but she has no say in the family

Since it is his first year, he does not know where to find help and support. He is not aware of the services that are available to him.

MAP NEEDS
- Not just clinical/counseling services but resources specifically for LGBT youth as well
- Low barrier to access map and good descriptions of locations to encourage using the services

54560 / 11-2017
