# Supplementary material for: The Optimization of an eHealth Solution (Thought Spot) with Transition-Aged Youth in Postsecondary Settings: Participatory Design Research
Source: J Med Internet Res. 2018 Mar 6;20(3):e79. doi: 10.2196/jmir.8102 (PMC5861299; doi:10.2196/jmir.8102)
Supplement: Multimedia Appendix 3 [file jmir_v20i3e79_app3.pdf]

## Appendix 3

***Coding matrix of topics discussed during Thought Spot workshops***

| **Topic** | **Sub-topic** |
|---|---|
| Appearance of Thought Spot | Layout |
| | Colours |
| | Consistency |
| Purpose of Thought Spot | Mental health |
| | Wellness |
| | Scope |
| Customization | Favourites |
| | Recommendations |
| | Personalization |
| Usability | Glitches |
| | Confusing |
| | Search |
| | Hard to use |
| | Easy to use |
| | Navigation |
| | Tutorial |
| Data | Categories/filters |
| | Ratings |
| | Description of services |
| | Verification |
| | Missing information |
| | Partnerships |
| | Crowd-sourcing |
| | Sustainability |
